# sEMG-Based Muscle Synergy Analysis and Functional Driving Ratio for Quantitative Assessment During Robot-Assisted Upper-Limb Rehabilitation

**DOI:** 10.3390/s26061952

**Published:** 2026-03-20

**Authors:** Baitian Tan, Jiang Shao, Qingwen Xu, Sujiao Li, Hongliu Yu

**Affiliations:** 1 Institute of Intelligent Rehabilitation Engineering, University of Shanghai for Science and Technology, No. 516 Jungong Road, Shanghai 200093, China; tim1097384919@163.com (B.T.);; 2 Shanghai Rehabilitation Assistive Device Engineering Technology Research Center, Shanghai 200093, China

**Keywords:** stroke, muscle synergy analysis, Functional Driving Ratio (FDR), robot-assisted upper limb rehabilitation, surface electromyography (sEMG)

## Abstract

Surface electromyography (sEMG) provides a non-invasive measure of the neural drive transmitted from the central nervous system to muscles by capturing the spatiotemporal summation of motor unit action potentials at the skin surface, and is therefore widely used to study neuromuscular coordination during motor tasks. By reflecting neural drive transmitted from the central nervous system to peripheral muscles, sEMG provides valuable insights for investigating neuromuscular coordination during upper-limb motor tasks. Within the framework of modular motor control, muscle synergy analysis has been increasingly applied to characterize coordinated muscle activation patterns extracted from multi-channel sEMG recordings. In this study, sEMG signals were collected from twelve stroke patients and nine healthy subjects during robot-assisted upper-limb training, involving two movement trajectories (straight and rectangular) and multiple robot-assisted levels. Muscle synergies were extracted using non-negative matrix factorization (NMF). A synergy merging–splitting model, combined with a Functional Driving Ratio (FDR), was employed to characterize both the muscle synergy reorganization and the relative activation contributions of driving versus stabilizing muscle components in terms of motor control strategy. The results showed that healthy subjects maintained consistent muscle coordination patterns across different assistive levels, while making task-dependent adjustments to muscle activation to adapt to variations in movement trajectories. For stroke patients, higher functional status was correlated with more differentiated coordination patterns and relatively higher FDR values, suggesting greater reliance on task-relevant agonist muscles during movement execution. In contrast, lower-function patients exhibited less differentiated coordination patterns accompanied by reduced FDR values, indicating the increased involvement of stabilizing or antagonist muscles. This shift may reflect compensatory control strategies and the reduced efficiency of neuromuscular coordination during assisted upper-limb movements. These findings suggest that sEMG-based muscle synergy features and the FDR may provide quantitative, sensor-derived support for characterizing neuromuscular coordination during robot-assisted rehabilitation.

## 1. Introduction

Stroke is an acute cerebrovascular event characterized by sudden onset and rapid progression. It often leads to impaired neural transmission along descending motor pathways within the central nervous system (CNS), resulting in abnormal muscle activation patterns and motor dysfunction [1,2,3,4]. A thorough understanding of CNS functional organization and motor control strategies is essential for elucidating the mechanisms underlying these impairments. How the CNS coordinates with the musculoskeletal system to support task-oriented movements in stroke patients remains a central topic in motor neuroscience and stroke rehabilitation research [5]. Recent advances in wearable sensors and surface electromyography (sEMG) have enabled the fine-grained monitoring of neuromuscular activity during human–robot interaction tasks [6]. However, translating raw sEMG signals into quantitative indicators of motor coordination remains challenging. The muscle synergy theory provides a practical framework for interpreting how the CNS mediates complex movements via low-dimensional control to reduce motor redundancy [7,8], offering insight into how the CNS organizes the musculoskeletal system into simplified control modules for movement execution. It has been applied in exercise science, control algorithms, and motion recognition [9,10,11,12], with studies (e.g., [13]) confirming that human motor behaviors rely on synergy-based low-dimensional control rather than independent muscle regulation. Accordingly, developing sEMG-based quantitative indicators to reliably characterize neuromuscular coordination during robot-assisted rehabilitation is critical for sensor-driven rehabilitation assessment and control. However, commonly used synergy-based metrics primarily describe coordination patterns and provide limited insight into the functional roles of individual muscles during task execution.

In rehabilitation medicine, repetitive and guided movement training has been proven to strengthen the musculoskeletal system and promote cortical repair and motor function recovery through neural plasticity. Rehabilitation robots, characterized by user-friendly interaction and low labor demand, have been widely adopted in post-stroke rehabilitation and have shown positive effects on neural reorganization. Based on these advantages, upper-limb rehabilitation robots have been increasingly used to investigate cortico-muscular coupling under various training environments. Studies by He et al. [14] found that healthy individuals exhibit consistent muscle synergy structures across three different trajectories, although relative muscle activation ratios and flexor contributions vary between trajectories. Similarly, Sadaka-Stephan et al. [15] reported that in robot-assisted passive reaching tasks, straight trajectories promote the formation of more consistent muscle organizations during active phases. Moreover, healthy individuals show stable intermuscular coordination strategies across tasks, suggesting a high degree of CNS consistency in regulating muscle coordination [16]. However, such consistency-focused commonly used synergy features, such as synergy number and similarity, primarily describe coordination patterns at a structural level and do not explicitly reflect the functional roles of individual muscles during task execution. This consistency forms the basis for comparing muscle synergy features between healthy and post-stroke populations, allowing the degree of motor dysfunction and neural reorganization to be inferred. Previous studies have shown that the severity of limb impairment in stroke patients is closely associated with time-invariant synergy features—such as synergy number, structure, and recruitment patterns. Muscle synergy analysis simplifies complex multi-muscle activations into low-dimensional components, providing a valuable perspective for understanding CNS regulation of muscle coordination. Although these features cannot directly represent cognitive neural states, time-invariant synergy parameters have been used as physiological indicators for cortical damage assessment and can complement clinical scales to characterize coordination impairments after stroke. In particular, abnormal muscle coordination patterns are closely related to clinical manifestations such as spasticity and impaired voluntary control, which can be quantitatively reflected by changes in muscle synergy organization.

Spasticity, a hallmark of impaired motor coordination, has been extensively studied using synergy analysis. Maistrello et al. [17] found that spasticity gradually decreases with post-stroke recovery when assessed by the Modified Ashworth Scale (MAS) and Brunnstrom stages, while Routson et al. [18] reported that stroke patients exhibited an increased number of muscle synergies and improved locomotor modularity after 12 weeks of gait training. Roh et al. [19] further revealed that abnormal muscle activation patterns in stroke patients correlate with their motor impairment levels, and Camardella et al. [20] observed that improvements in Fugl–Meyer Assessment (FMA) and Action Research Arm Test (ARAT) scores were associated with changes in synergy characteristics—suggesting that motor recovery may result from forming new task-oriented control strategies rather than simply restoring normal physiological patterns.These findings suggest that synergy reorganization reflects functional adaptation rather than direct recovery of normal physiological patterns. Accordingly, stroke recovery involves both functional adaptive strategy formation and approximation to healthy coordination patterns; while full restoration of physiological synergies is not required for functional recovery, multi-trajectory and repetitive training can drive muscle synergy reorganization toward healthy-like structural characteristics. Therefore, comparing muscle synergy parameters between stroke patients and healthy controls is crucial for understanding the mechanisms underlying stroke-induced motor impairments. However, existing synergy-based metrics mainly describe coordination patterns and provide limited insight into how muscles functionally contribute to movement execution under different assistive conditions.

In this study, we propose a quantitative framework for evaluating post-stroke upper-limb motor coordination based on multi-channel sEMG-driven muscle synergy analysis during robot-assisted rehabilitation. Surface electromyography (sEMG) signals were collected from healthy subjects performing upper-limb robotic training at Assistive Levels 1 and 3, and from stroke patients performing at Assistive Level 3, under both straight and rectangular trajectories. Muscle synergies were extracted using non-negative matrix factorization, and a synergy merging–splitting model combined with the Functional Driving Ratio (FDR) was introduced to quantify the balance between driving and stabilizing muscle contributions. Rather than representing muscle recruitment strength, FDR serves as a functional proxy that reflects the relative contribution of driving and stabilizing muscle components during task execution. By constructing reference synergies from healthy subjects under different assistive conditions, the similarity between patient and healthy synergy patterns was systematically analyzed to explore how coordination patterns vary across functional levels and assistive contexts [21,22]. The proposed framework is intended to provide objective, sensor-based quantitative support for the analysis of neuromuscular coordination in robot-assisted post-stroke rehabilitation, with a focus on potential applications in assessment support and training adjustment.

## 2. Experimental Methods

### 2.1. Participants

A total of 14 stroke patients in the recovery phase (Brunnstrom stages II–V) and 9 healthy subjects were recruited for this study. A total of 2 stroke patients were excluded due to data loss caused by EMG electrode detachment, resulting in 12 stroke patients being included in the final analysis. Stroke participants were recruited from inpatients and outpatients at Shanghai First Rehabilitation Hospital between February and November 2023. The stroke group consisted of two males and ten females, aged 44–74 years (mean ± SD: 62.50 ± 11.71 years). Among them, seven patients presented with left-sided hemiplegia and five with right-sided hemiplegia.

All healthy participants were right-handed and performed the same experimental tasks as the stroke group. No statistically significant differences in demographic characteristics were observed between the two groups (*p* > 0.05), as summarized in Table 1.

The inclusion criteria for stroke patients were as follows:

1. Diagnosis consistent with the Diagnostic Criteria for Various Cerebrovascular Diseases in China (2019) [23] and confirmed by cranial CT or MRI;
2. Age between 25 and 80 years;
3. No gender restriction;
4. Clear consciousness without severe cognitive impairment, with the ability to understand and follow instructions during rehabilitation training and assessment;
5. Disease duration between 2 weeks and 6 months (recovery phase);
6. Full passive range of motion in the affected upper limb.

The exclusion criteria were as follows:

1. Brunnstrom stage I;
2. Severe cognitive, behavioral, or language dysfunction (MMSE < 14).

Before enrollment, stroke patients were assessed by experienced clinicians and classified into two subgroups according to upper limb Brunnstrom stages: the low-function group (stages II–III) and the high-function group (stages IV–V). This study was conducted in accordance with the Declaration of Helsinki and was approved by the Ethics Committee of Shanghai First Rehabilitation Hospital (Approval No.: YK-2023-01-003). Written informed consent was obtained from all participants prior to inclusion.

### 2.2. Experimental Setup

To ensure precise control of movement trajectories and standardized task execution, the ArmGuider upper limb rehabilitation robot, jointly developed by Shanghai Zhuodao Medical Technology Co., Ltd., Shanghai, China, and the Intelligent Rehabilitation Research Institute, University of Shanghai for Science and Technology, was used as the experimental platform [24,25], thereby maintaining consistent kinematic constraints across experimental conditions.

**Figure 1:**
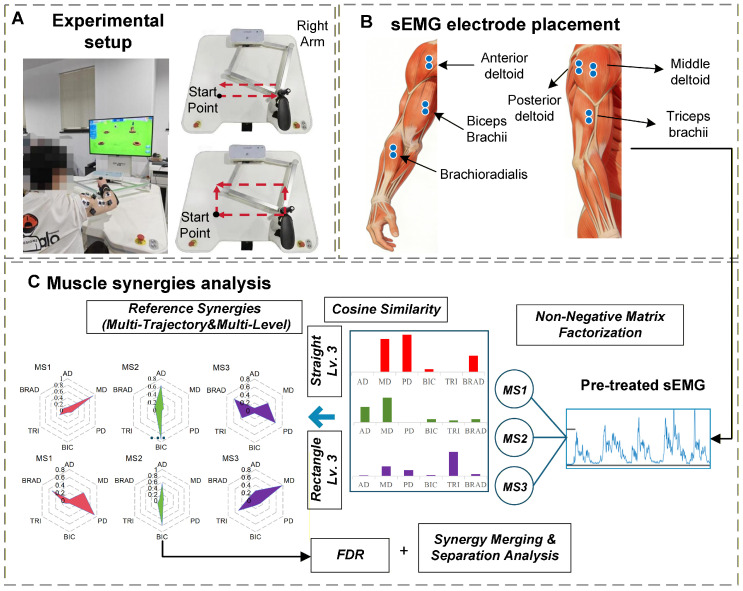
The overall workflow of the current study. (**A**) Images of task set-up and training trajectories. (**B**) Acquisition of sEMG. (**C**) Muscle synergy extraction using the NMF algorithm and evaluation of patient synergies based on reference synergies from healthy subjects.

The ArmGuider system is an end-effector–based upper-limb rehabilitation robot designed to provide planar movement assistance in a horizontal workspace. During rehabilitation training, the robot constrains the end-effector to predefined trajectories while allowing voluntary force generation along the movement direction. Robot assistance was implemented in a force-based manner, in which the robot provided supportive force along the target trajectory when the participant’s voluntary output was insufficient. Higher assistive levels corresponded to greater robotic force contribution, thereby reducing the muscular effort required for task execution. Conversely, lower assistive levels required increased voluntary force generation from the participant while maintaining the same kinematic constraints. Importantly, while the movement trajectory was constrained by the robot, participants retained the ability to modulate muscle activation patterns and force output within the assisted framework. This design allowed the investigation of how changes in task demand induced by different assistance levels influence muscle coordination strategies, rather than altering the movement goal itself. Participants sat comfortably with their forearm securely fixed to the robotic arm and manipulated the arm along two predefined trajectories (straight and rectangular) within an interactive game scenario. As illustrated in Figure 1A, a virtual hammer displayed on the screen was used to strike gophers located at the trajectory endpoints. A directional arrow indicated the required force direction and was updated after each successful hit to cue the subsequent movement, forming a continuous task sequence.

In the assisted mode, the robot guided the movement when voluntary effort was insufficient. A scoring mechanism was used to encourage active participation. When the participant’s contribution exceeded 20% of the total movement force, 10 points were awarded; otherwise, 1 point was given.

All healthy participants were right-handed and performed the task with their dominant arm, while stroke patients used their affected arm.

The rectangular trajectory consisted of four sequential phases:

1. Pushing vertically forward;
2. Pushing outward;
3. Pulling backward;
4. Returning horizontally to the start, forming a cyclic motion.

The straight trajectory included two phases:

1. Pushing horizontally from the proximal to distal point;
2. Returning to the start position.

**Figure 2:**
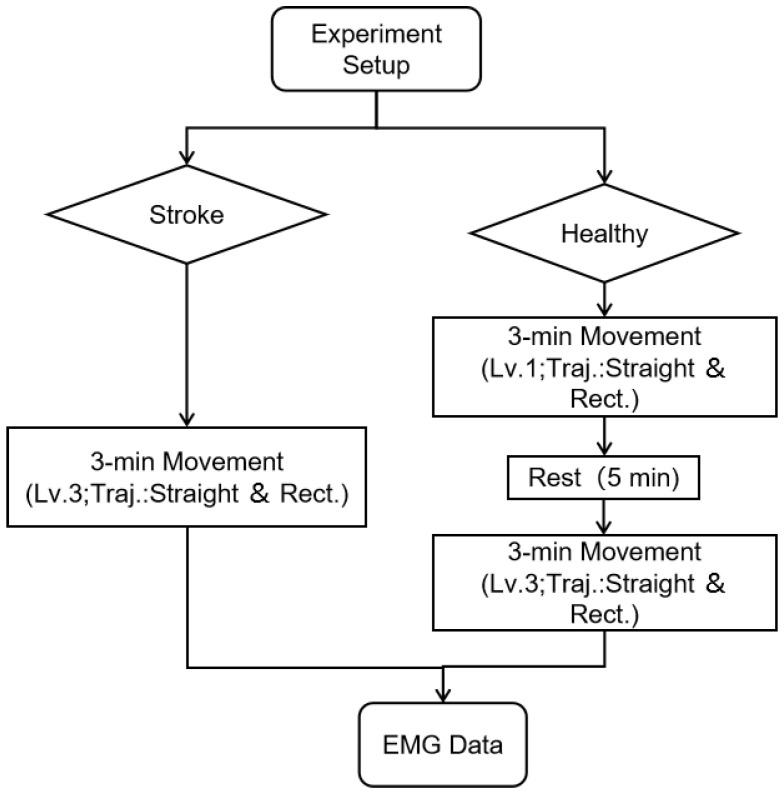
The process of collecting electromyographic signals of the subjects.

Healthy participants first completed 3 min of training at assistive level 1, followed by a 5 min rest, and then 3 min of training at assistive level 3 (assistive level 1: low assistance; assistive level 3: high assistance). Stroke patients, due to limited upper limb control, performed only 3 min of training at assistive level 3 along both trajectories, as illustrated in Figure 2.

### 2.3. Data Acquisition

Multi-channel sEMG signals were acquired at a sampling frequency of 1500 Hz using the Noraxon Ultium system (Noraxon USA Inc., Scottsdale, AZ, USA), a high-precision, multi-mode wireless system for upper limb movement analysis. Signals were recorded from six key upper-limb muscles involved in the experimental tasks, including the anterior deltoid (AD), middle deltoid (MD), posterior deltoid (PD), biceps brachii (BIC), triceps brachii (TRI), and brachioradialis (BRAD), as shown in Figure 1B.

**Figure 3:**
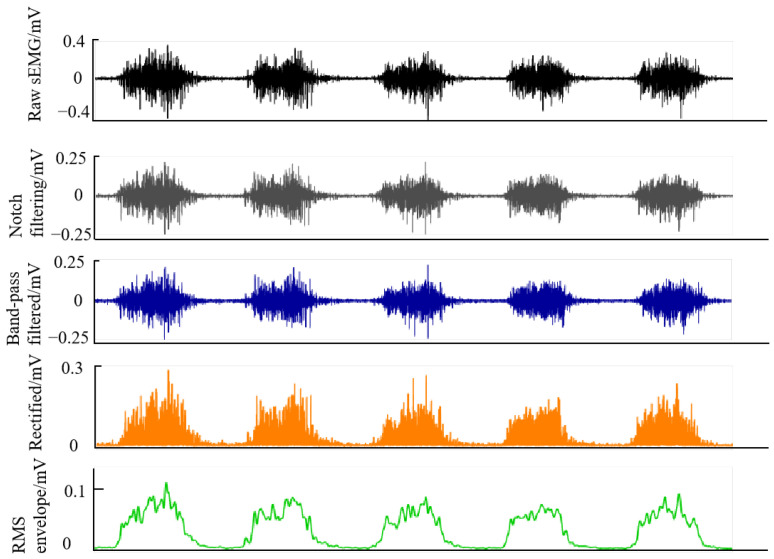
Representative sEMG signal from a single muscle channel illustrating the standard preprocessing steps, including the raw signal, 50 Hz notch-filtered signal, 20–300 Hz band-pass-filtered signal, full-wave rectified signal, and RMS-smoothed signal. This preprocessing pipeline was used to suppress noise and electrical interference, as well as to enhance signal quality to support subsequent muscle synergy analysis.

Before electrode placement, the skin at each target site was prepared by hair removal and cleaning with ethanol to reduce skin impedance and improve electrode–skin contact. The raw sEMG signals were preprocessed using a standard pipeline to suppress noise and interference and to improve data quality for subsequent analyses. The preprocessing steps included the following, as shown in Figure 3:

1. A 50 Hz notch filtering for power-line interference removal;
2. A 20–300 Hz band-pass filtering to retain physiological signal components;
3. Full-wave rectification;
4. Root mean square (RMS) smoothing.

## 3. Methods

### 3.1. Data Analysis and Muscle Synergy Extraction

To characterize neuromuscular coordination under different trajectories and assistance levels, surface electromyography (sEMG) signals were collected during each experimental trial. For consistency across conditions and computational efficiency, each trial was resampled to t=14,000 time points. Muscle synergies were then extracted using non-negative matrix factorization (NMF), which decomposes multichannel sEMG data into spatial and temporal components under a non-negativity constraint.

Let $V\in R^{m\times t}$ denote the preprocessed sEMG matrix, where m=6 represents the number of recorded muscles and t=14,000 denotes the number of time samples. NMF approximates *V* as the product of two non-negative matrices:

(1) V≈WH,W≥0,H≥0

where $W\in R^{m\times r}$ is the muscle weighting matrix encoding the relative contribution of each muscle to each synergy, and $H\in R^{r\times t}$ is the activation coefficient matrix describing the temporal modulation of the *r* synergies. The parameter *r* represents the number of muscle synergies and reflects the dimensionality of motor control.

Since the number of motor modules underlying central nervous system (CNS) control is not known a priori, an objective criterion is required to determine the appropriate number of synergies. The Variance Accounted For (VAF) was used to evaluate the quality of reconstruction provided by the synergy model.

The VAF is defined as

(2) $$\mathrm{VAF}=1-\frac{\sum {\left(V_{\mathrm{original}}-V_{\mathrm{reconstructed}}\right)}^{2}}{\sum {\left(V_{\mathrm{original}}-{\bar{V}}_{\mathrm{original}}\right)}^{2}}$$

where $V_{\mathrm{original}}$ and $V_{\mathrm{reconstructed}}$ denote the original and reconstructed sEMG signals, respectively, and ${\bar{V}}_{\mathrm{original}}$ represents the mean of the original data.

The number of synergies was increased incrementally until the overall VAF exceeded 90% [26]. If adding an additional synergy resulted in a VAF improvement of less than 5%, the lower-dimensional solution was selected. This empirically adopted criterion balances reconstruction accuracy and physiological interpretability and has been widely used in previous synergy studies.

### 3.2. Synergy Similarity Analysis

To evaluate the consistency of muscle synergy structures across healthy individuals and quantify stroke-related alterations, the same number of synergies was extracted from each participant. Synergies derived from healthy subjects were treated as reference synergies.

Cosine similarity was used to quantify the similarity between two synergy vectors, $w_{i}$ and $w_{j}$:

(3) $$\mathrm{Similarity}=\frac{w_{i}\cdot w_{j}}{∥w_{i}∥\;∥w_{j}∥}$$

A similarity value greater than 0.8 was considered indicative of high pattern similarity, suggesting that the corresponding synergies represent comparable neuromuscular coordination patterns under the same movement condition.

### 3.3. Synergy Merging and Splitting Analysis

Following stroke, cortical lesions disrupt normal neural regulation, leading to alterations in the modular organization of muscle synergies. Based on the framework proposed by Zhang et al. [15,27], three patterns may be observed: synergy preservation, synergy merging, and synergy splitting. Synergy merging occurs when a patient synergy can be reconstructed as a non-negative linear combination of multiple reference synergies:

(4) $$S_{i}^{\left(p\right)}\approx \sum_{k=1}^{N_{c}}a_{ik},S_{k}^{\left(r\right)},\;a_{ik}\geq 0$$

This phenomenon reflects a reduced differentiation among synergy components, indicating a simplified coordination pattern compared to healthy reference synergies. To identify meaningful merging relationships, only coefficients exceeding a predefined threshold (0.2) were considered to contribute to synergy merging, thereby excluding weak or potentially noisy contributions.

Conversely, synergy splitting is observed when a reference synergy is reconstructed from multiple patient synergies:

(5) $$S_{k}^{\left(r\right)}\approx \sum_{i=1}^{N_{s}}b_{ki},S_{i}^{\left(p\right)},\;b_{ki}\geq 0$$

Synergy splitting indicates increased fragmentation of synergy representations relative to healthy reference patterns, reflecting altered coordination strategies. Similarly, only coefficients larger than 0.2 were regarded as indicative of effective synergy splitting.

### 3.4. Functional Driving Ratio (FDR)

While synergy merging and splitting describe changes in synergy composition, they do not explicitly capture the functional balance between force generation and joint stabilization during movement execution. To address this limitation, a Functional Driving Ratio (FDR) was introduced as a functionally interpretable index derived from synergy muscle weightings. The FDR is defined as

(6) $$\mathrm{FDR}=\frac{W_{\mathrm{driver}}}{W_{\mathrm{driver}}+W_{\mathrm{stabilizer}}}$$

where $W_{\mathrm{driver}}$ and $W_{\mathrm{stabilizer}}$ represent the summed normalized weights of muscles classified as driving and stabilizing components within each synergy, respectively. The FDR quantifies the relative contribution of task-relevant agonist muscles to movement execution in comparison with muscles primarily involved in joint stabilization or postural control.

A higher FDR indicates a greater relative contribution of driving muscle components within a synergy, suggesting a coordination strategy that emphasizes task-directed force generation. In contrast, a lower FDR reflects the increased relative involvement of stabilizing or antagonist muscles, which may be associated with compensatory coordination strategies commonly observed after stroke. It should be noted that the FDR does not represent muscle activation magnitude or neural recruitment strength, nor does it directly measure underlying neural mechanisms. Instead, the FDR serves as a functional proxy that facilitates the interpretation of muscle synergy organization in terms of movement-driving and stabilizing roles under different task and assistance conditions. In this study, the anterior deltoid (AD) and middle deltoid (MD) were identified as primary driving muscles responsible for shoulder flexion and horizontal abduction, respectively. The posterior deltoid (PD) was considered a phase-dependent driving muscle and was excluded from the core driving group, because its contribution varied across movement phases and trajectories, making it less suitable as a consistent driving muscle indicator across tasks. The biceps brachii (BIC) and brachioradialis (BRAD) primarily contributed to elbow flexion and posture maintenance, whereas the triceps brachii (TRI) functioned as an antagonist stabilizer. These muscles were therefore classified as stabilizing muscles.

### 3.5. Statistical Analysis

Given the relatively small sample size and the non-Gaussian distribution of the extracted synergy-related metrics, non-parametric statistical tests were employed throughout the study. Specifically, the Mann–Whitney U test was used for between-group comparisons, including comparisons of the number of extracted muscle synergies between healthy subjects and stroke patients (as well as between low-function (LF) and high-function (HF) stroke subgroups), comparisons of intergroup differences in synergy similarity values, and comparisons of intergroup differences in Functional Driving Ratio (FDR) between LF and HF stroke patients under straight and rectangular trajectories. All statistical tests were two-tailed, and a significance level of *p* < 0.05 was adopted. Statistical analyses were performed using MATLAB (R2019a, MathWorks, Natick, MA, USA).

## 4. Results

### 4.1. Number of Muscle Synergies

**Figure 4:**
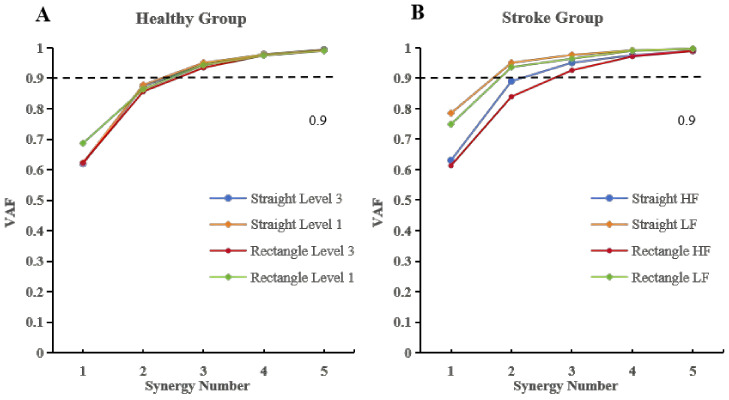
Number of muscle synergies under two trajectories. (**A**) VAF variation in twelve healthy subjects in the control group under two assisting levels. (**B**) VAF variation in nine patients with HF and LF group under power level 3. The dotted line indicates the VAF threshold of 90% used to determine the number of muscle synergies.

**Figure 5:**
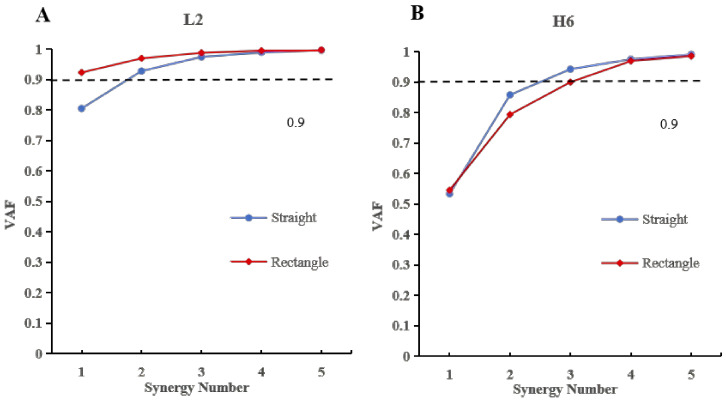
Examples of synergy merging and splitting patterns observed in representative patients from the LF and HF subgroups. (**A**) L2. (**B**) H6. The dotted line indicates the VAF threshold of 90% used to extract muscle synergies.

The number of muscle synergies was quantified for each subject under different trajectory types and assistance levels. Synergy number was determined separately for straight and rectangular trajectories based on the VAF criterion. In healthy subjects, the number of synergies was highly consistent across trajectories and assistance levels. For both straight and rectangular movements, the overall VAF exceeded 90% when three synergies were extracted, regardless of assistive level (Figure 4A). In the stroke group, four patients were classified into the low-function (LF) subgroup and eight into the high-function (HF) subgroup (Figure 4B). All LF patients exhibited reduced synergy numbers, ranging from one to two, consistent with a pronounced reduction in synergy dimensionality. Consistent with the group-level reduction in synergy number observed in the LF subgroup, a representative LF subject (L2) exhibited two synergies during straight movements and only one synergy during rectangular movements, illustrating a pronounced synergy merging pattern (Figure 5A). Similarly, consistent with the higher synergy numbers observed in the HF subgroup, a representative HF subject (H6) showed a synergy number comparable to healthy controls during straight movements and exhibited four synergies during rectangular movements, illustrating a synergy splitting pattern (Figure 5B).

Group-level statistical analysis showed that, under the straight trajectory, the mean number of synergies in the stroke group (2.25 ± 0.60) was lower than that of the healthy group at assistive level 1 (2.68 ± 0.47) and level 3 (2.68 ± 0.47), although no significant differences were observed (p>0.05). Similarly, under the rectangular trajectory, the mean synergy number in stroke patients (2.75 ± 0.72) was lower than that of healthy subjects at level 1 (2.67 ± 0.82) and level 3 (3.11 ± 0.57), without statistical significance (p>0.05). Within the stroke group, subgroup analysis revealed that for the straight trajectory, the LF subgroup exhibited fewer synergies (1.75 ± 0.43) than the HF subgroup (2.50 ± 0.50), although the difference was not statistically significant (p>0.05). For the rectangular trajectory, the LF subgroup exhibited a lower number of synergies (2.00 ± 0.71) compared with the HF subgroup (3.12 ± 0.33, p=0.015), suggesting more differentiated synergy structures in patients with higher motor function.

### 4.2. Extraction of Muscle Synergies in the Healthy Group

**Figure 6:**
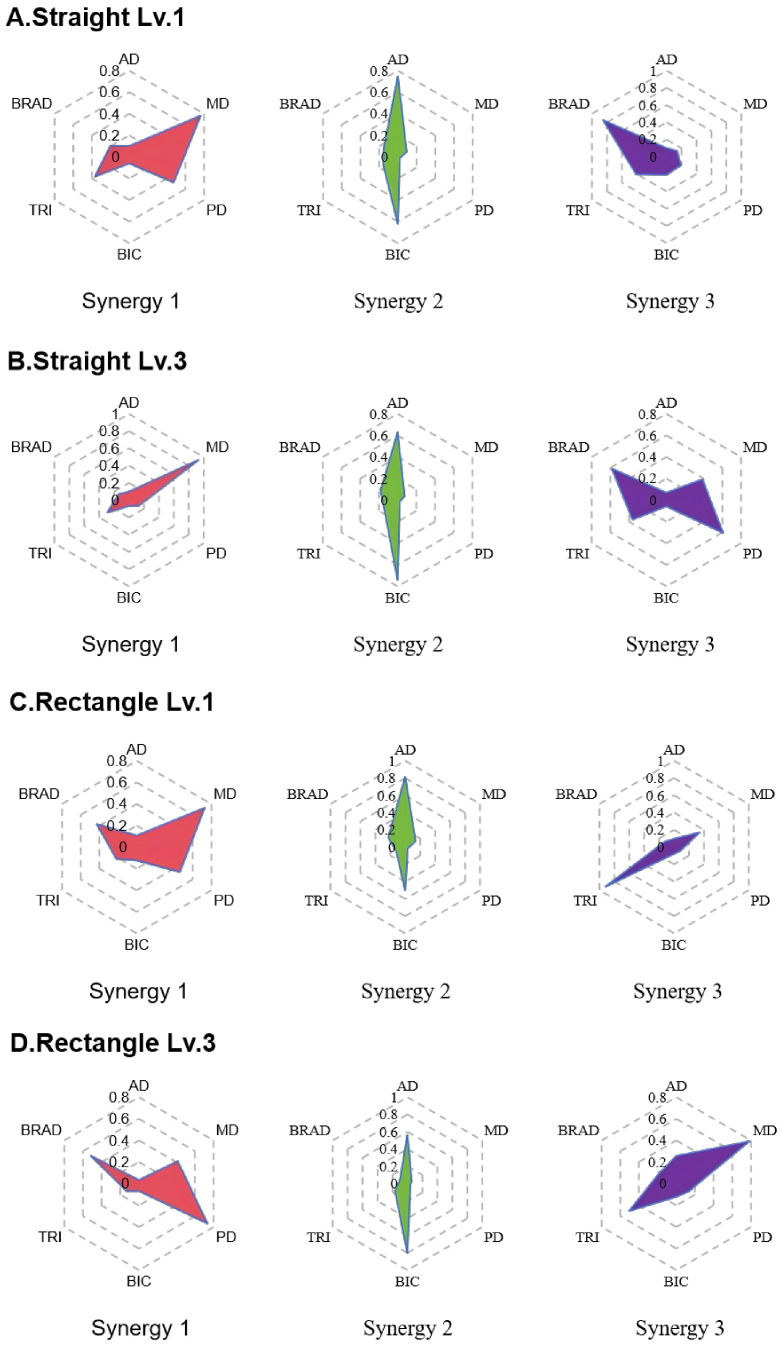
Group-level reference muscle synergies of the healthy group represented as radar plots under different trajectories and assistance levels. (**A**) Straight trajectory with level 1 assistance. (**B**) Straight trajectory with level 3 assistance. (**C**) Rectangular trajectory with level 1 assistance. (**D**) Rectangular trajectory with level 3 assistance.

Minor inter-individual variability was observed in the number of extracted muscle synergies among healthy participants. For both straight and rectangular trajectories, the overall VAF exceeded 0.90 when three synergies were extracted. Therefore, the number of synergies was fixed at r=3 for all healthy subjects to establish a uniform reference. After synergy vector matching within the healthy group, cosine similarity was computed between corresponding synergy vectors. The mean similarity values were 0.84 ± 0.15 for the straight trajectory with assistive level 3, 0.88 ± 0.09 for the straight trajectory with assistive level 1, 0.83 ± 0.13 for the rectangular trajectory with assistive level 3, and 0.87 ± 0.11 for the rectangular trajectory with assistive level 1, indicating high intra-group consistency of synergy structures. Based on these consistent synergy patterns, group-level reference muscle synergies for the healthy group were constructed by averaging synergy matrices across the four experimental conditions. The resulting reference synergy vectors are illustrated using radar plots in Figure 6.

Across both trajectory types and assistance levels, three characteristic muscle synergy patterns were identified in healthy subjects, each exhibiting distinct muscle weighting distributions. For the straight trajectory, Synergy 1 was dominated by the middle deltoid, with additional contributions from the posterior deltoid under lower assistance. Synergy 2 showed prominent activation of the anterior deltoid and biceps brachii, while Synergy 3 was characterized by higher contributions from the brachioradialis, with secondary involvement of the triceps brachii. For the rectangular trajectory, Synergy 1 primarily involved the middle and posterior deltoids. Synergy 2 exhibited a muscle composition similar to that observed in the straight trajectory, dominated by the anterior deltoid and biceps brachii. Synergy 3 showed increased weighting of the triceps brachii.

### 4.3. Comparison of Synergy Parameters Between Two Groups

**Figure 7:**
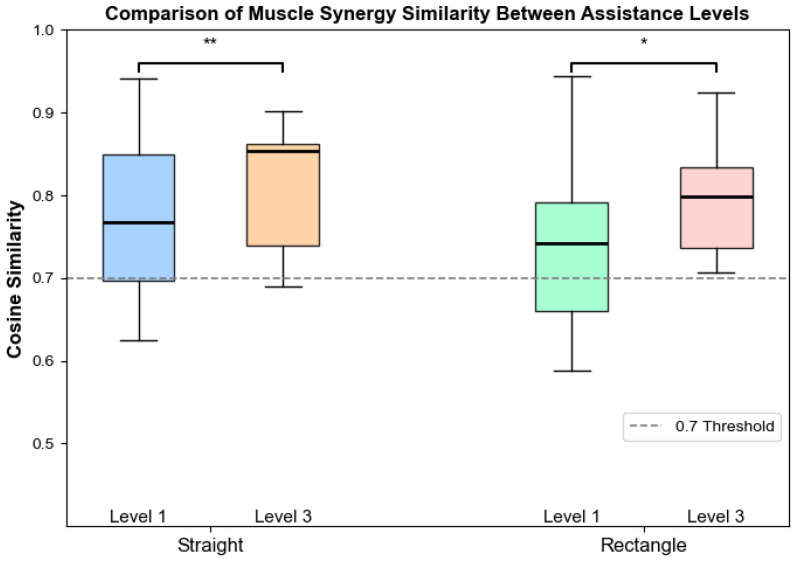
Comparison of cosine similarity between muscle synergies of stroke patients and reference synergies of healthy subjects under different assistive levels and movement trajectories. Four boxplots represent the following conditions: straight trajectory at assistive level 1 and level 3, and rectangular trajectory at assistive level 1 and level 3. Significant differences between assistive levels within the same trajectory are indicated by asterisks (* p<0.05, ** p<0.01). The dashed horizontal line denotes the cosine similarity threshold of 0.7.

Figure 7 illustrates the cosine similarity between patient muscle synergies under assistive level 3 and the reference synergies of healthy subjects under different assistance levels. To examine the association between robotic assistance level and muscle synergy structure in stroke patients, cosine similarity was calculated between patient synergies under assistive level 3 and the reference synergies of healthy subjects under different assistance levels.

For the straight trajectory, the similarity between patient synergies under level 3 assistance and healthy reference synergies under level 3 assistance (0.65 ± 0.09) was significantly higher than that with reference synergies under level 1 assistance (0.61 ± 0.07, p=0.0012). For the rectangular trajectory, a similar trend was observed. The similarity with healthy reference synergies under level 3 assistance (0.78 ± 0.09) was significantly higher than that with level 1 reference synergies (0.71 ± 0.09, p=0.0261).

### 4.4. Group-Level FDR Characteristics and Representative Synergy Reorganization in Stroke Patients

**Figure 8:**
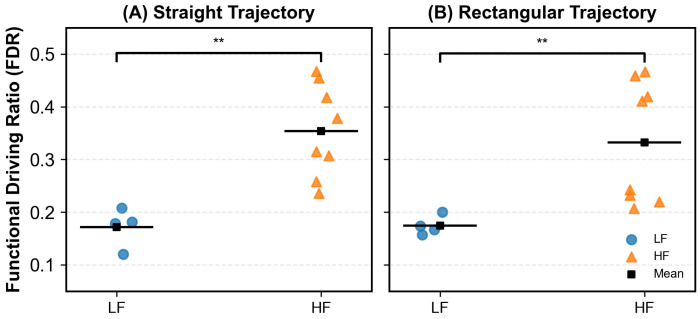
Group-level distribution of Functional Driving Ratio (FDR) in low-function (LF) and high-function (HF) stroke patients under (**A**) straight and (**B**) rectangular trajectories. ** indicates statistical significance at p<0.01.

To investigate whether the Functional Driving Ratio (FDR) exhibits distinct intergroup patterns, we compared the FDR distributions of low-function (LF) and high-function (HF) stroke patients under straight and rectangular trajectories (Figure 8). As shown in Figure 8, HF patients exhibited significantly higher FDR values than LF patients across both trajectories. At the group level, the mean FDR of LF patients was 0.172 for the straight trajectory and 0.174 for the rectangular trajectory, whereas HF patients showed higher mean FDR values of 0.354 and 0.332, respectively. These intergroup differences reached statistical significance for both trajectories (*p* = 0.004), suggesting a relatively greater contribution of task-relevant driving muscles to motor execution in patients with higher motor function. Notably, substantial inter-individual variability was observed within both subgroups, as evidenced by the dispersed distribution of individual data points.

**Figure 9:**
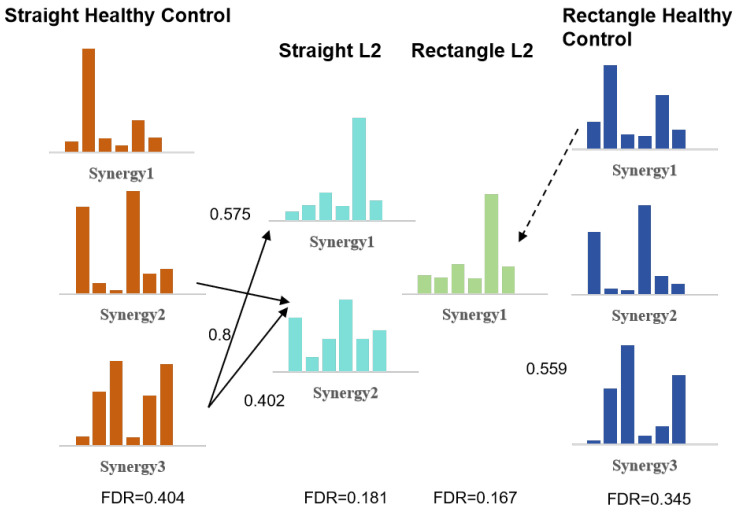
Synergy merging results for Patient L2. The middle synergy vectors represent the extracted synergies under straight and rectangular trajectories, respectively. The synergy vectors on both sides correspond to the group-level reference synergies of healthy subjects under assistive level 3. Arrows indicate effective correspondence between patient-specific and healthy reference synergies based on the synergy merging and separation model (coefficients > 0.2).

To further illustrate the underlying synergy reorganization mechanisms associated with these group-level trends, representative cases from the LF and HF subgroups were analyzed. Based on the synergy merging and splitting model described in Equations (4) and (5), representative results of muscle synergy reorganization are illustrated for one low-function patient (L2) and one high-function patient (H6). Figure 9 shows the synergy merging results for Patient L2. The synergy vectors on both sides represent the reference synergies derived from healthy subjects under straight and rectangular trajectories, respectively. The middle synergy vectors correspond to the synergies extracted from Patient L2 under the same trajectory conditions.

For the straight trajectory, two synergy vectors were identified for Patient L2. Patient Synergy 2 was reconstructed as a non-negative combination of healthy reference synergy 2 and healthy reference synergy 3, with corresponding merging coefficients of 0.575 and 0.800. The resulting synergy pattern showed a higher weighting of the biceps brachii (BIC). Patient Synergy 1 exhibited a muscle weighting distribution similar to that of healthy reference synergy 3 and was primarily characterized by higher weighting of the triceps brachii (TRI). For the rectangular trajectory, only one synergy vector was extracted. Its muscle weighting pattern was comparable to that observed in Patient Synergy 1 under the straight trajectory and was mainly characterized by activation of the triceps brachii (TRI). The Functional Driving Ratio (FDR) values of Patient L2 were 0.181 for the straight trajectory and 0.167 for the rectangular trajectory.

**Figure 10:**
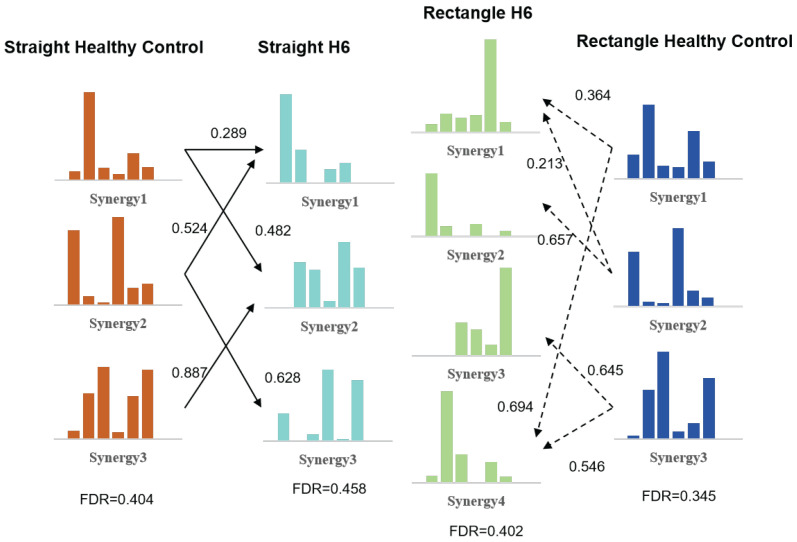
Synergy splitting results for Patient H6. The middle synergy vectors represent the extracted synergies under straight and rectangular trajectories, respectively. The synergy vectors on both sides correspond to the reference synergies of healthy subjects under assistive level 3. Arrows indicate effective correspondence between patient-specific and healthy reference synergies based on the synergy merging and separation model (coefficients > 0.2).

Figure 10 presents the synergy splitting results for Patient H6. The middle synergy vectors represent the synergies extracted under straight and rectangular trajectories, respectively, while the side vectors correspond to the healthy reference synergies. For Patient H6, multiple patient synergies contributed to the reconstruction of individual healthy reference synergies, consistent with a pronounced synergy splitting phenomenon. The FDR values for Patient H6 were 0.458 under the straight trajectory and 0.402 under the rectangular trajectory.

## 5. Discussion

### 5.1. Physiological Characteristics of Muscle Coordination After Stroke

Stroke-induced damage to the corticospinal tract disrupts descending neural commands, resulting in impaired regulation of motor neuron pools and abnormal muscle activation patterns [28]. Muscle synergy analysis provides a physiologically meaningful framework for characterizing such deficits by revealing how the central nervous system (CNS) organizes muscles into low-dimensional control modules to support coordinated movement execution [29,30,31,32]. In the present study, healthy subjects consistently exhibited three muscle synergies across trajectories once the Variance Accounted For (VAF) exceeded 90%, indicating stable and modular neuromuscular control. In contrast, stroke patients demonstrated a reduced number of synergies, reflecting diminished motor complexity and impaired modular organization. Notably, patients in the low-function (LF) subgroup exhibited fewer synergies than those in the high-function (HF) subgroup, with a statistically significant difference observed under the rectangular trajectory condition.

These findings suggest that reductions in synergy number are associated with the severity of motor impairment. Rather than representing a simple loss of muscle activity, fewer synergies indicate a collapse of independent motor modules into simplified control patterns. This observation is consistent with previous studies reporting synergy reduction as a hallmark of impaired neural regulation following stroke [27,30,33]. Therefore, synergy number may serve as a quantitative indicator of motor control degradation and may reflect the functional status of post-stroke neuromuscular coordination.

### 5.2. Establishment of Reference Synergies

To ensure reliable evaluation of patient muscle coordination, group-level reference synergies were established using healthy subjects under task-matched conditions. Two planar trajectories were examined: a straight trajectory involving primarily horizontal motion, and a rectangular trajectory incorporating both horizontal and vertical components. Distinct muscle activation patterns were observed between trajectories, indicating that synergy structure is task dependent rather than invariant across movement conditions [34,35]. In addition to trajectory effects, assistance level was associated with alterations in muscle recruitment patterns in healthy individuals. Although the overall number of synergies in healthy subjects remained unchanged, the distribution of muscle weightings across synergies varied with assistance level. These assistance-dependent changes in muscle weighting likely reflect alterations in task-level demands on movement-driving and stabilizing muscles, providing a theoretical rationale for using assistance-matched reference synergies in subsequent analyses. For example, posterior deltoid contributions shifted between synergy components when assistance increased, suggesting that robotic support alters the balance between driving and stabilizing muscle roles [36,37].

Because stroke patients were only able to complete training under assistive Level 3, synergy similarity analysis demonstrated that patient synergies were significantly more similar to healthy reference synergies obtained under the same assistance level than to those derived under lower assistance. This finding highlights the importance of assistance-matched reference synergies for accurate interpretation of patient muscle coordination patterns. Accordingly, reference synergies derived from healthy subjects under Level 3 assistance were adopted as reference synergies in subsequent analyses. This strategy minimizes confounding effects introduced by task and assistance mismatches and provides a physiologically meaningful baseline for evaluating post-stroke motor coordination.

### 5.3. Functional Interpretation of Synergy Reorganization Using FDR

While synergy number and structural similarity provide insight into modular organization, they do not directly capture the functional balance between force generation and joint-level stabilization. To address this limitation, the Functional Driving Ratio (FDR) was introduced as a physiologically interpretable index derived from synergy muscle weightings. FDR quantifies the relative contribution of task-relevant driving muscles versus stabilizing muscles within each synergy. At the behavioral level, FDR reflects how the central nervous system allocates control emphasis between movement-driving and stabilizing functions across different motor strategies and impairment levels. Lower FDR values indicate a relative overreliance on stabilizing or antagonist muscle activity, whereas higher FDR values reflect efficient recruitment of agonist muscles responsible for movement execution. Unlike purely mathematical similarity measures, FDR directly links synergy structure to functional motor output. This interpretation is in line with recent studies emphasizing relative muscle contribution rather than activation magnitude alone [38,39], making FDR particularly suitable for interpreting post-stroke motor deficits. In the present study, this functional interpretation of FDR was supported by clear group-level differences between stroke subgroups. High-function (HF) patients exhibited significantly higher FDR values than low-function (LF) patients across both straight and rectangular trajectories, indicating a greater relative contribution of task-relevant driving muscles to movement execution in patients with better motor function. These findings suggest that FDR captures clinically relevant differences in motor control efficiency at the group level, beyond structural descriptors of synergy organization alone.

### 5.4. Case-Level Interpretation of Synergy Merging and Splitting

Representative cases from the LF and HF subgroups were analyzed to illustrate distinct patterns of synergy reorganization following stroke. In the LF patient (L2), pronounced synergy merging was observed across both trajectories, accompanied by consistently low FDR values. The marked reduction in independent driving-related synergies, particularly those dominated by the middle deltoid, indicates reduced motor selectivity and inefficient force generation. This pattern suggests that multiple independent control modules have collapsed into a single synergy dominated by stabilizing muscles. Such synergy merging is commonly interpreted as a compensatory strategy adopted by the CNS following injury, whereby motor control is simplified at the expense of efficiency and adaptability. This phenomenon has been reported in previous studies and is considered a characteristic feature of severe motor impairment [40,41]. In contrast, the HF patient (H6) exhibited clear synergy splitting, with multiple patient synergies contributing to the reconstruction of individual healthy reference synergies. This splitting was accompanied by higher FDR values approaching those of healthy subjects, indicating improved recruitment of driving muscles and a more balanced coordination strategy, although these patterns did not fully reach the coordination characteristics observed in healthy subjects. Synergy splitting in this context may reflect the emergence of compensatory neural pathways or partial restoration of modular control, enabling more flexible and efficient movement execution.

Together, these findings suggest that synergy merging and splitting represent distinct modes of post-stroke motor reorganization. When interpreted in conjunction with FDR, they provide complementary structural and functional insights into neuromuscular recovery processes. Although phase- or direction-specific analyses may reveal finer temporal modulation, the present condition-level FDR captures global motor control strategies associated with functional impairment and assistance level.

### 5.5. Limitations

Several limitations of this study should be acknowledged. Firstly, given the relatively small sample sizes of both healthy subjects and stroke patients, the present work should be regarded as exploratory, aiming to evaluate the feasibility and sensitivity of sEMG-based muscle synergy features and the Functional Driving Ratio (FDR) in robot-assisted upper-limb rehabilitation rather than to draw definitive or generalizable conclusions. Secondly, although standardized experimental protocols were employed, inherent variability in voluntary motor execution and insufficient movement consistency across participants and trials are common in neuromuscular studies involving active tasks, which may have influenced the extracted sEMG signals and derived synergy characteristics. Future studies with larger cohorts and more rigorous control of movement repeatability are therefore warranted to further validate and extend the proposed framework. Despite these limitations, the present results demonstrate the potential of combining muscle synergy analysis with FDR to provide physiologically meaningful insights into post-stroke neuromuscular coordination during robot-assisted rehabilitation.

## 6. Conclusions

This study proposed a quantitative framework for characterizing post-stroke neuromuscular coordination during upper-limb rehabilitation by integrating muscle synergy analysis with the Functional Driving Ratio (FDR). Group-level reference synergies were established from healthy subjects under task- and assistance-matched conditions, enabling reliable comparison of muscle coordination patterns between healthy individuals and stroke patients. The results showed that stroke patients exhibited reduced synergy dimensionality and altered synergy organization, reflecting impaired modular motor control. Synergy merging and splitting analysis revealed distinct patterns of motor reorganization associated with different functional levels. Furthermore, the FDR provided a physiologically interpretable metric to quantify the balance between driving and stabilizing muscle contributions within each synergy, offering functional insight beyond structural similarity measures alone.

Overall, the proposed framework enables a multidimensional evaluation of neuromuscular control by jointly considering synergy structure, reorganization behavior, and functional muscle contributions. This approach provides an objective and interpretable tool for assessing motor impairment and recovery after stroke and has the potential to support individualized rehabilitation assessment and inform the design and adjustment of robot-assisted training strategies. Moreover, this work provides a foundation for sensor-driven, personalized adjustment of robotic assistance in future rehabilitation systems.

## Figures and Tables

**Table 1:** Post-Stroke Patients’ Information of LF and HF Groups.

Subj	Sex	Age	Affected Side	Lovett Grading	Hand Brunnstrom Grading
Low-Function (LF) Group					
L1	M	56	L	II	II
L2	M	74	R	II	II
L3	M	45	R	IV	III
L4	M	80	L	III	III
High-Function (HF) Group					
H1	M	68	L	III	IV
H2	M	63	R	V	IV
H3	F	56	R	II	V
H4	F	71	L	IV	III
H5	M	53	L	V	IV
H6	M	74	L	IV	IV
H7	M	66	L	IV	IV
H8	M	44	R	IV	V

Note: LF = low function; HF = high function; M = male; F = female; L = left (affected upper limb side); R = right (affected upper limb side).

## Data Availability

Data are contained within the article.
